# Supplementary material for: Unraveling Size Dependent Bi‐ and Tri‐Exciton Characteristics in CdSe/CdS Core/Shell Quantum Dots via Ensemble Time Gated Heralded Spectroscopy
Source: Small. 2025 Nov 17;22(1):e09793. doi: 10.1002/smll.202509793 (PMC12757981; doi:10.1002/smll.202509793)
Supplement: Supplementary file 1 — Supporting Information [file SMLL-22-e09793-s001.pdf]

NANO · MICRO
small

Supporting Information

for *Small*, DOI 10.1002/smll.202509793

Unraveling Size Dependent Bi- and Tri-Exciton Characteristics in CdSe/CdS Core/Shell Quantum Dots via Ensemble Time Gated Heralded Spectroscopy

Einav Scharf, Rotem Liran, Adar Levi, Omer Alon, Nadav Chefetz, Dan Oron and Uri Banin**

Supporting Information

Unraveling Size Dependent Bi- and Tri-exciton Characteristics in CdSe/CdS Core/Shell Quantum Dots via Ensemble Time Gated Heralded Spectroscopy

Einav Scharf[1], Rotem Liran[1], Adar Levi[1], Omer Alon[1], Nadav Chefetz[1], Dan Oron[2], Uri Banin[1*]*

[1]Institute of Chemistry and the Center for Nanoscience and Nanotechnology, The Hebrew University of Jerusalem, Jerusalem 91904, Israel

[2]Department of Molecular Chemistry and Materials Science, Weizmann Institute of Science, Rehovot 7610001, Israel

[*]Corresponding authors

Email: uri.banin@mail.huji.ac.il, dan.oron@weizmann.ac.il

Table of contents

Section S1. Supplementary figures 3
Figure S1 3
Figure S2 4
Figure S3 4
Figure S4 5
Figure S5 6
Figure S6 6
Figure S7 7
Figure S8 8
Figure S9 9
Figure S10 10
Figure S11 10
Figure S12 11
Figure S13 12
Figure S14 12
Figure S15 13
Figure S16 13
Figure S17 14
Figure S18 15
Figure S19 15
Section S2. Supplementary text 16
Table S1 16
References 17

Section S1. Supplementary figures

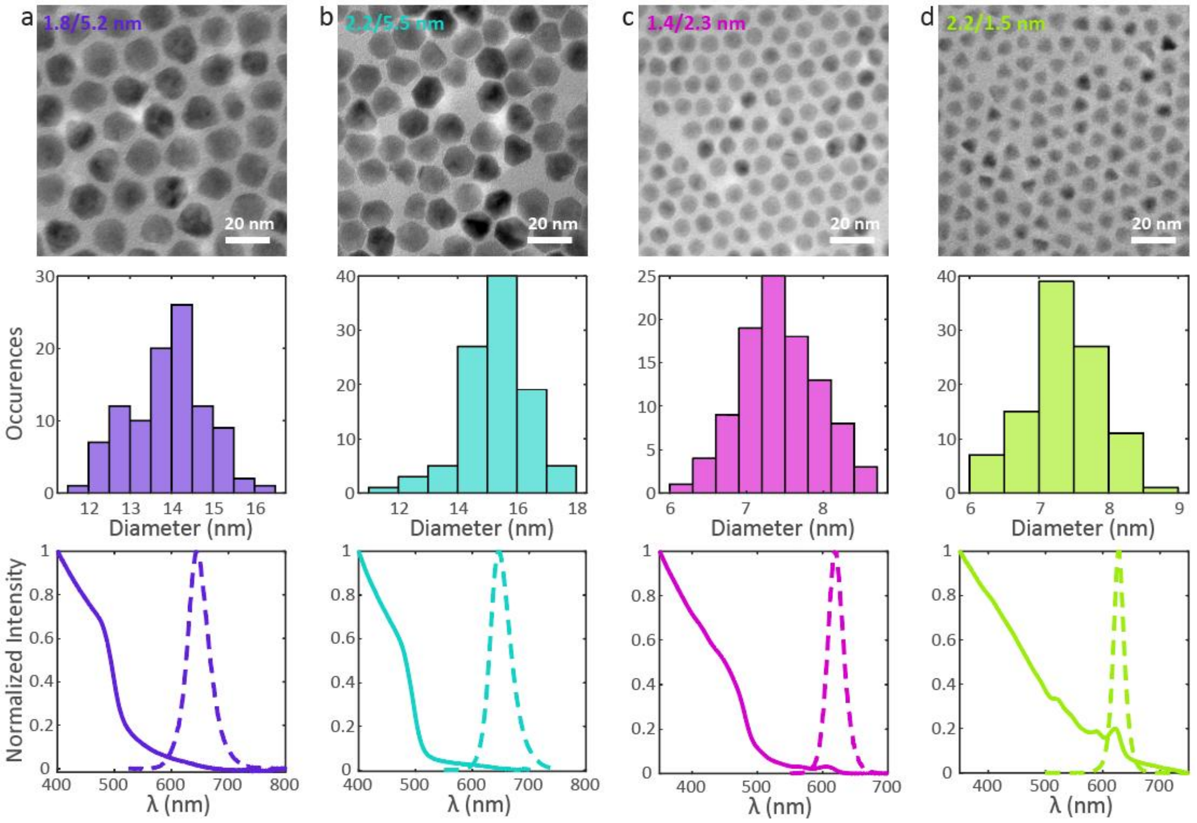

Figure S1. Size distributions of the studied quantum dots. The top panels present the transmission electron microscope images of the quantum dots (QDs) that were used in this study. The sizes stated on the top panels showcase the core radii and shell thicknesses. The middle panels present the size distributions with average diameters and standard deviations of (a) 14.0±0.9, (b) 15±1, (c) 7.4±0.5, (d) 7.4±0.5 nm. The bottom panels present the absorption and emission spectra (in solid and dashed lines, respectively). The full width at half maximum is (a) 125, (b) 118, (c) 90, (d) 66 meV.

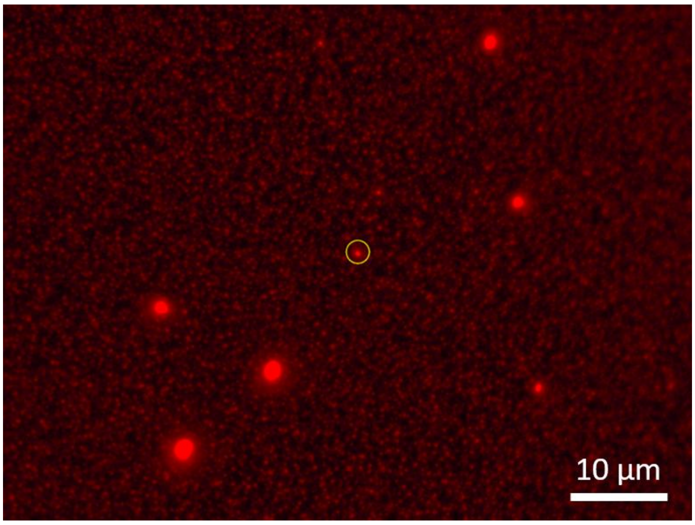

Figure S2. Widefield photoluminescence image of 1.8/5.2 nm CdSe/CdS QDs. A widefield photoluminescence image presenting the dispersion of QDs and QD aggregates on the glass substrate. The bright spots are QD aggregates. The yellow circle marks an aggregate that was measured in this study.

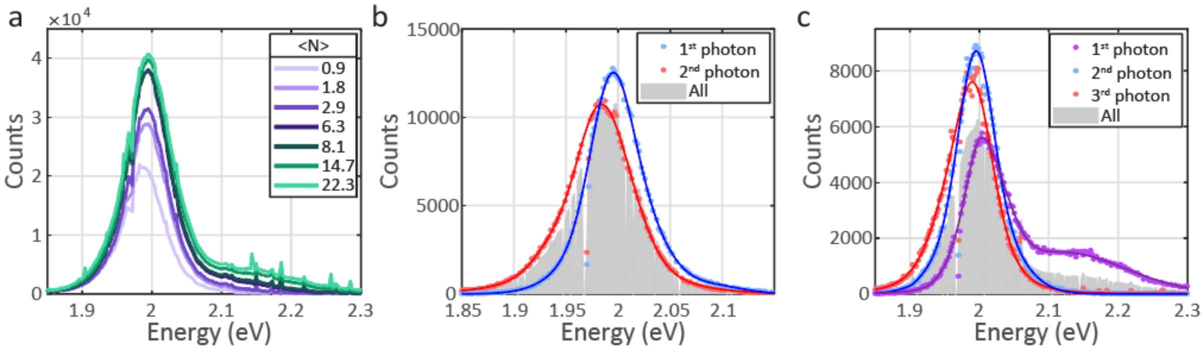

Figure S3. Characterization of the aggregate in Figure 1g in the main text. (a) Power dependent spectrum of the aggregate. The $\langle N \rangle$ values were calculated similarly to the values in Figure 2e in the main text. (b) Spectral characterization of the photon pair events at $\langle N \rangle = 0.9$. The blue dots represent the first photons in the pairs and the pink dots represent the second photons in the pairs. They are both fitted to a multi-gaussian model (in blue and red, respectively). The gray area is the normalized spectrum of all photon events. (c) Spectral characterization of the photon triplet events at $\langle N \rangle = 22.3$. The first, second, and third photons are in purple, blue, and pink dots, respectively, and are fitted to a multi-gaussian model. The gaps in the spectra are due to excluded noisy pixels. The temporal conditions for the arrival times of the photons in panels b and c are as stated in the Experimental section in the main text.

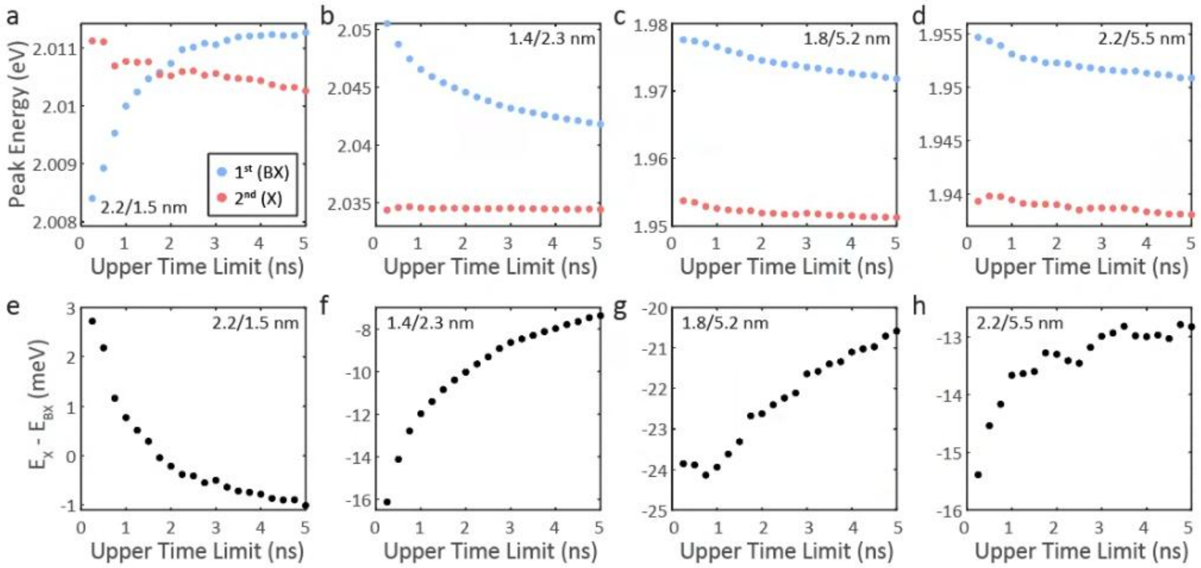

Figure S4. (a)-(d) Peak energies of the first (blue) and second (pink) photons within the photon pair events versus the upper limit of the time gate of the first photons (the lower limit is t=0) in the various core/shell sizes that were studied. The second photons are restricted to arrive after the first photons and up to 200 ns after the arrival of the first photons. (e)-(h) are the energy differences between the second and first photons versus the upper time limit for the first photons in the various studied samples. This analysis showcases that applying a short time gate for the arrival of the first photons in the pairs helps to increase the relative contribution of biexcitons (BXs), apparent by the higher BX energy in short times (or lower energy in the case of the 2.2/1.5 nm QDs, where the BX is red shifted). The time gate presented in the main text is 1 ns. Choosing this short time gate was especially important for the 2.2/1.5 nm QD sample, as with a time gate higher than 1.75 ns the attractive nature of the X-X interaction is masked. On the other hand, this analysis shows that small changes in the choice of the temporal gate do not change the energies significantly (by few meV at most). The reported BX energy in the main text is extracted from the first data point of this analysis (the energy of the first photons, arriving in the first 0.25 ns after the laser pulse). The error values, presented for the BX binding energies in the main text, reflect the minor influence of this selection, among other contributions.

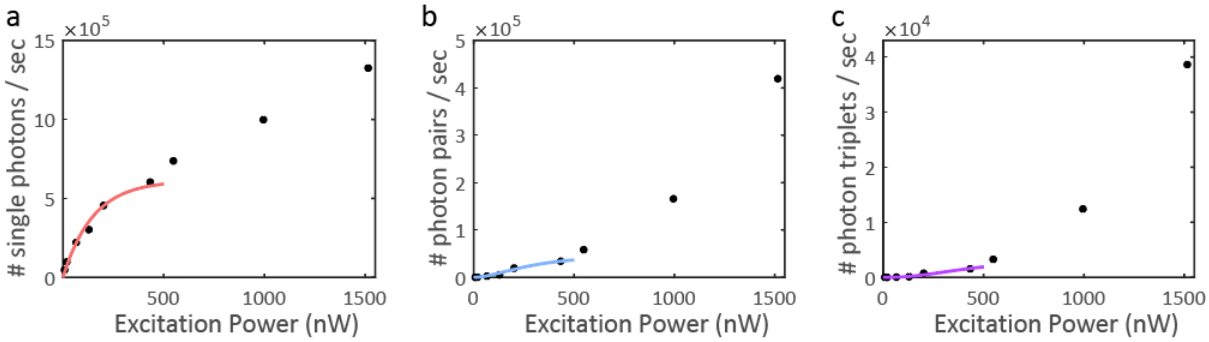

Figure S5. The number of single photon events, photon pair events, and photon triplet events per second versus excitation power in panels a, b, and c, respectively (in black dots). The colored lines are fits to the probability to generate at least one, two, or three excitons (in pink, blue, and purple, respectively) according to the Poisson distribution. This is the same analysis as in Figure 2e in the main text, yet it presents the points in the three higher excitation powers that were neglected in the main text. This sharp incline in the emission intensity is due to transition to the quasi-continuous-wave regime, in which the multi-excited QDs emit rapidly during the laser pulse.[1]

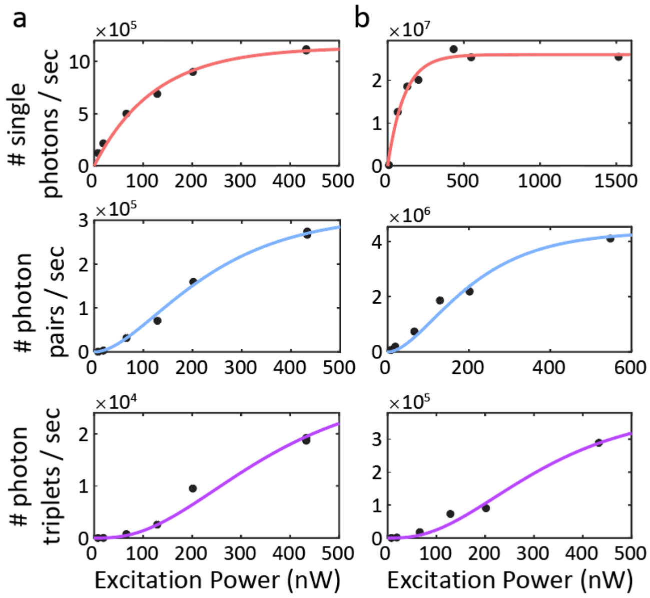

Figure S6. Examples of saturation curves of two 1.8/5.2 nm aggregates. (a) and (b) represent two different aggregates of QDs. The top, middle, and bottom panels present the number of single photon events, photon pair events, and photon triplet events per second versus excitation power. The colored lines are fits to the probability to generate at least one, two, or three Xs (pink, blue, and purple, respectively) according to the Poisson distribution. The

saturation powers are extracted from the top panels: (a) 120±20 nW ($R^2 = 0.991$), (b) 110±20 nW ($R^2 = 0.981$), and used in the fits of the middle and bottom panels, with R^2 of (a) 0.996, 0.978; (b) 0.974, 0.980, for the middle and bottom panels, respectively.

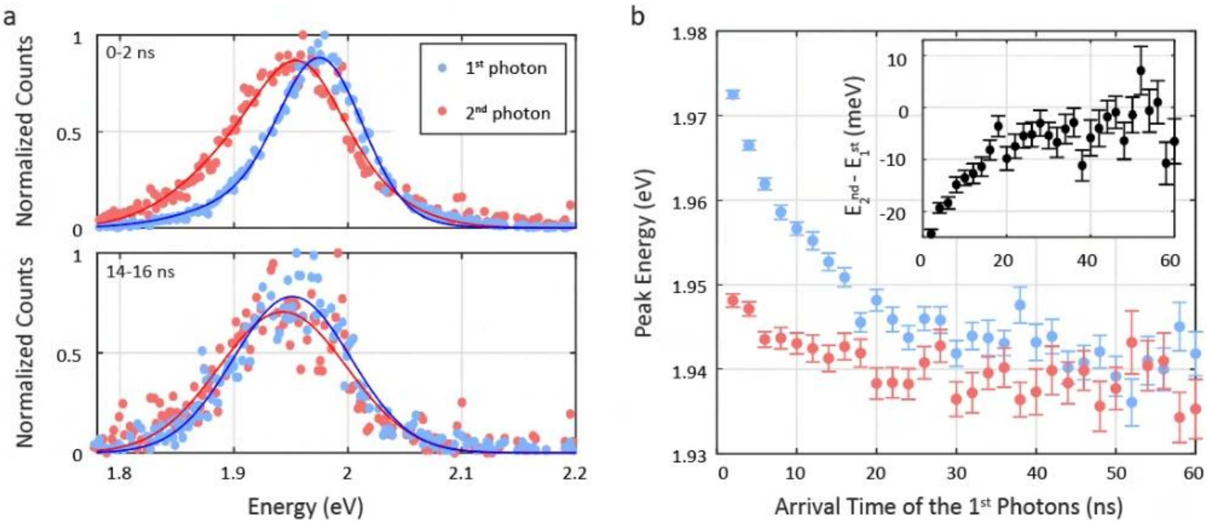

Figure S7. Biexciton time gated analysis. This analysis is for the same aggregate of QDs as in Figure 1h-j, 2-3, 5 in the main text, at $\langle N \rangle = 0.13 \pm 0.03$. (a) The spectrum of the first and second photons (in blue and pink, respectively) within the photon pair events. In the top (bottom) panel the arrival time of the first photons is set to 0-2 ns (14-16 ns), and the spectrum of the first photons exhibits a blue shift (no shift) relative to the spectrum of the second photons. The shift in short times represents the interaction within the BX state, whereas after 14 ns the majority of the BXs decayed, and the photon pair events include mostly uncorrelated single excitons (Xs) emitted from different QDs. The spectra are fitted to a multi-gaussian model. (b) Time-dependent peak energies of the spectrum of the first photons (in blue) and of the spectrum of the second photons (in pink), in bins of 2 ns for the arrival time of the first photons in the pairs (the Xs arrive later, between 0.8 and 200 ns after the first photons). The black dots in the inset are the difference between the peak energies of the first and second photons' spectra. The first photons (in blue) exhibit a significant red shift with time, due to the transition from BX-dominated emission to X-dominated emission. Notably, even in longer times, after the BXs decayed, the first photon is somewhat higher in energy than the second photon. This is assigned to the size dispersion in the ensemble, as generally smaller QDs emit faster and in higher energy than larger QDs.[2] Interestingly, the X emission peak in pink slightly red shifts with time as well. This observation is unlikely to stem from the BX emission. For this end, we followed the emission of a single QD in low excitation power (Figure S7).

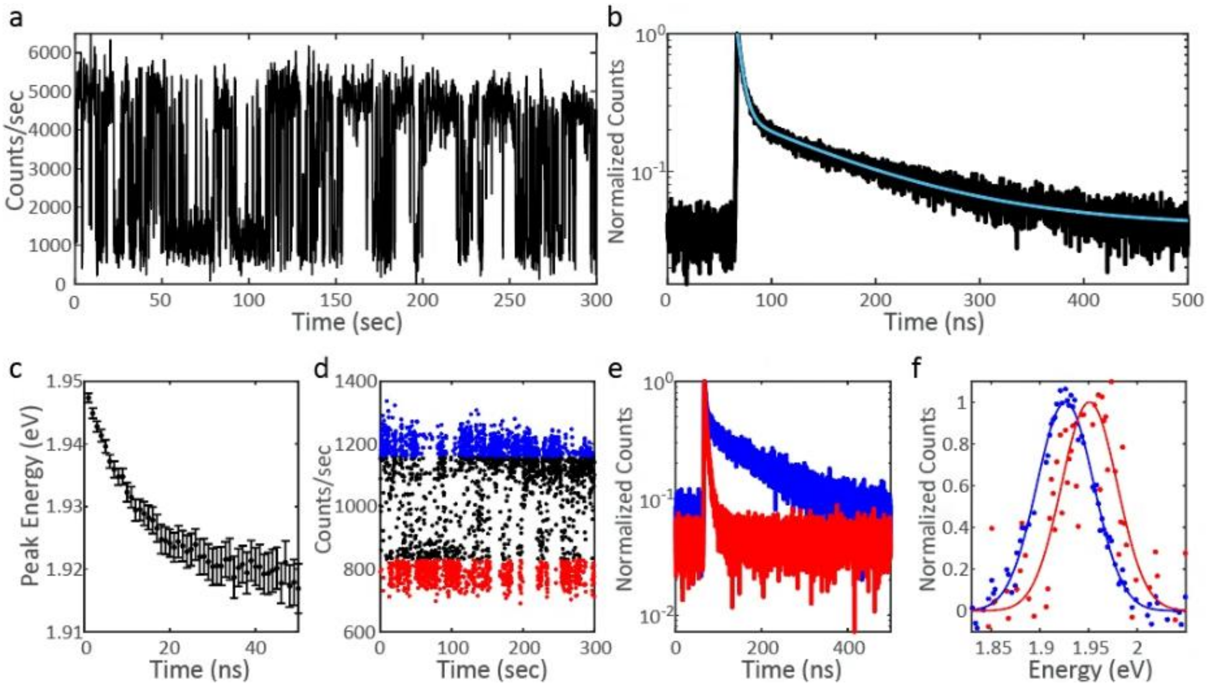

Figure S8. Characterization of a single QD. (a) Fluorescence intensity fluctuation time-trace of a single QD at $\langle N \rangle = 0.1$ (excited with an EPL450 pulsed laser by Edinburgh Instruments). The transition between an emissive "on" state and a non-emissive "off" state (i.e. blinking) is indicative of emission of single QDs.[3] (b) Fluorescence decay histogram fitted to a bi-exponential decay (in light blue), showcasing short and long lifetime components (5.3±0.1, 113±1 ns). (c) The peak energy of the emission spectrum versus the arrival time of the photons (in bins of 1 ns), showcasing a red shift in longer times. (d) To understand this behavior in the single QD, we studied the top 30% of the counts (i.e., the "on" state, in blue) and the bottom 30% of the counts (i.e., the "off" state, in red) within the fluorescence intensity fluctuation time-trace. The "on" state is usually associated with neutral X emission, whereas the "off" state is usually associated with charged Xs or even multiexcitons (MXs), that undergo Auger recombination.[4] (e) Fluorescence decay histograms of the "on" state (in blue) and of the "off" state (in red). The decay histogram of the "off" state exhibits only contribution of the short lifetime component, in line with Auger recombination of charged Xs. The decay histogram of the "on" state exhibits a dominant contribution of the long lifetime component, in line with neutral X emission. (f) Normalized emission spectra of the top 30% of the counts (in blue) and the bottom 30% of the counts (in red), fitted to a gaussian distribution. The emission peaks of the "on" and "off" states are 1.926±0.001 and 1.951±0.002, respectively. This indicates that the neutral X emission in a single QD is red shifted by ~25 meV than the charged X emission.

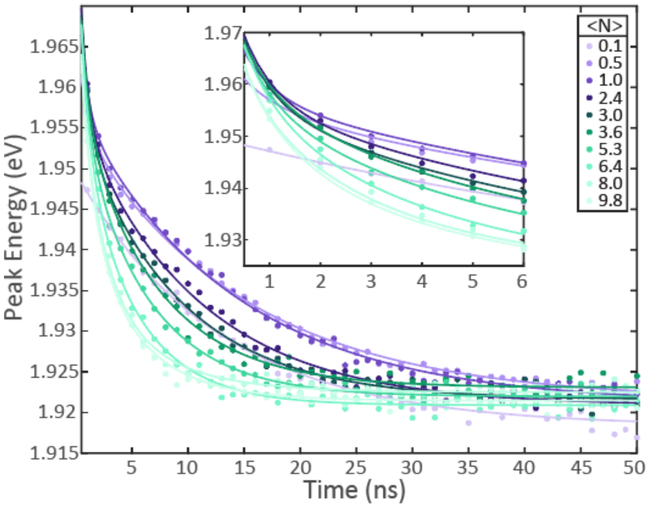

Figure S9. Power dependent energy shift with time in a single QD. The peak energy of the emission spectrum versus the arrival time of the photons (in bins of 1 ns) in various excitation powers, in the same single QD as in Figure S8. The $\langle N \rangle$ values are calculated according to the Poisson distribution (similarly to the analysis in Figure 2e in the main text). This power dependent representation of the red shift of the spectrum with time is crucial in order to fully understand the source of the shift. In Figure S8 the "off" state, with fast-emitting photons, is blue shifted relative to the later arriving photons. This could indicate contribution of Auger recombination of either charged Xs or MXs. This figure shows that in the low excitation power of $\langle N \rangle = 0.1$ (with its elaborated results in Figure S8) the peak energy in short times is 1.947 eV. The inset, which zooms in to the first 6 ns of this analysis clearly shows that when increasing the excitation power, a further blue shift is apparent in short times. For example, at $\langle N \rangle = 0.5$ the peak energy in short times is 1.957 eV. In conclusion, the further blue shift in higher excitation powers suggests BX emission (or emission of MXs of higher orders in high powers). However, at $\langle N \rangle = 0.1$ the probability to generate MXs is low, and thus the blue shift in short times is dominated by emission of charged Xs.

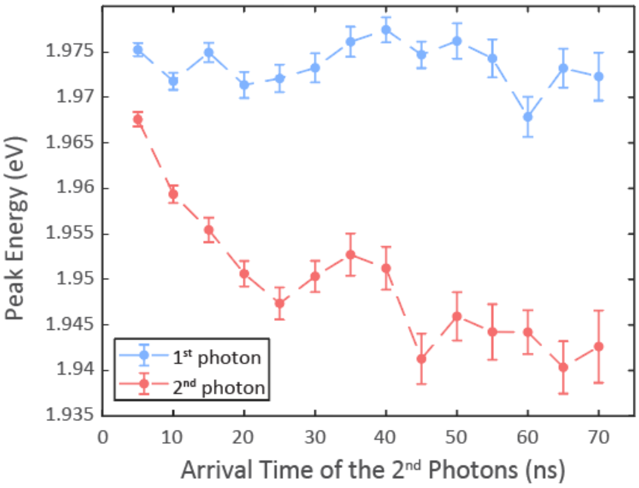

Figure S10. The peak energies of the spectrum of the first photons (in blue) and of the spectrum of the second photons (in pink), where the x axis represents the arrival time of the second photons in the pairs (in 5 ns bins). Accordingly, the energy of the first photons does not change significantly, as it includes only photons that arrive in the first 3 ns in all the data points. However, the emission peak of the second photons red shifts with time, due to the decreasing contribution of charged excitons in longer times. Following this analysis, the emission energy of the neutral X was determined as 1.942 eV, as the emission energy of the second photons in long arrival times.

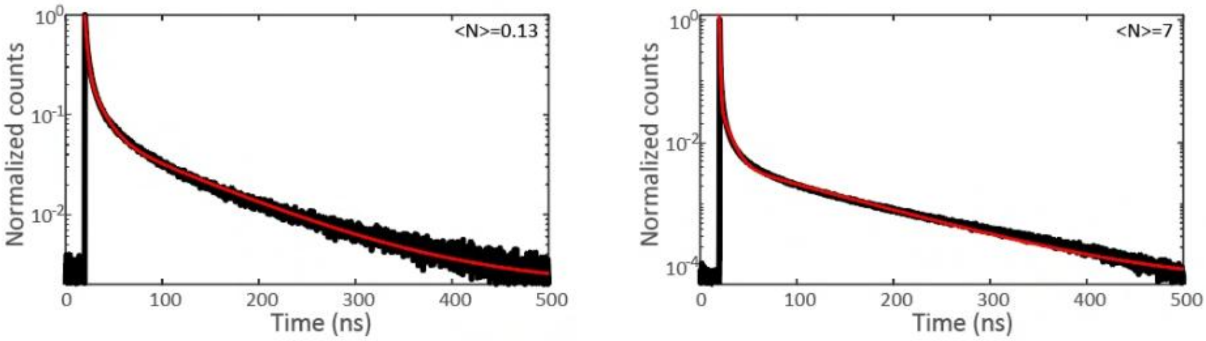

Figure S11. Fluorescence decay histograms at excitation powers of $\langle N \rangle = 0.13 \pm 0.03$ (left panel) and $\langle N \rangle = 7 \pm 2$ (right panel) of the same ensemble of QDs as in Figure 1h-j, 2-3, 5 in the main text. Each of the histograms are fitted to tri-exponential decays, where the long component represents the neutral X emission, the short component represents MX emission, and the intermediate component likely represents charged X emission.[5] The fitted lifetimes of the weakly excited aggregate (in the left panel) are 2.4±0.1, 13.8±0.2, 105±1 ns, and the fitted lifetimes of the strongly excited aggregate (in the right panel) are 0.70±0.01, 8±1, 100±45 ns.

The large error value in the long lifetime component is due to the low contribution of the long component to the decay behavior (less than 1%).

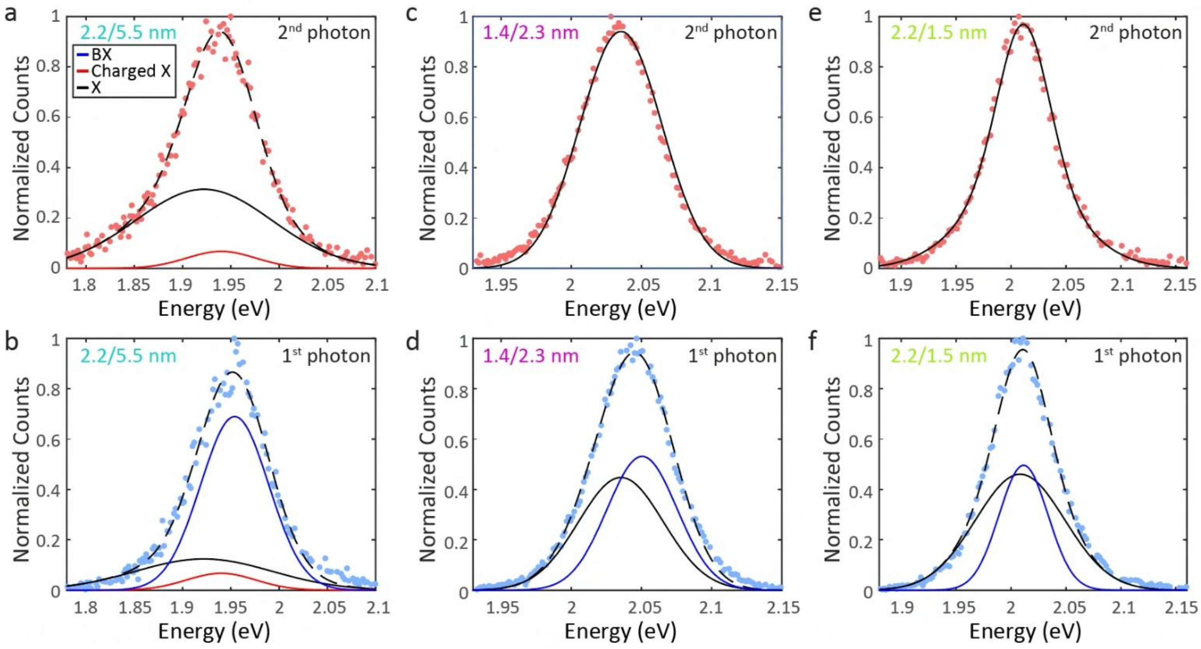

Figure S12. Size dependent biexciton analysis. The spectrum of the second and first photons (top panels in pink dots and bottom panel in blue dots, respectively) within the photon pair events for 2.2/5.5 nm QDs (in a, b), 1.4/2.3 nm QDs (in c, d), and 2.2/1.5 nm QDs (in e, f). Only pairs where the first photons arrived in the first 2 ns are included. The second photons in (a) are fitted to a sum of two gaussians (in a dashed black line). Its components represent neutral X emission (black line) and charged X emission (red line). Similarly, the first photons' spectrum in (b) is fitted to a sum of three gaussians (in a dashed black line). The black and red components are the same as in (a), representing neutral and charged X emission, respectively. The blue line represents BX emission. This analysis follows the same procedure of the 1.8/5.2 nm QDs presented in Figure 3 in the main text. The energies of the emitting states are 1.922 (X), 1.94 (charged X), and 1.954 eV (BX). For the smaller QDs in panels c-f fits of a single gaussian for the second photons and a sum of two gaussians for the first photons are sufficient. The energies of the emitting states in the 1.4/2.3 nm QDs (in c, d) are 2.035 (X), 2.051 eV (BX). The energies of the emitting states in the 2.2/1.5 nm QDs (in e, f) are 2.011 (X), 2.008 eV (BX).

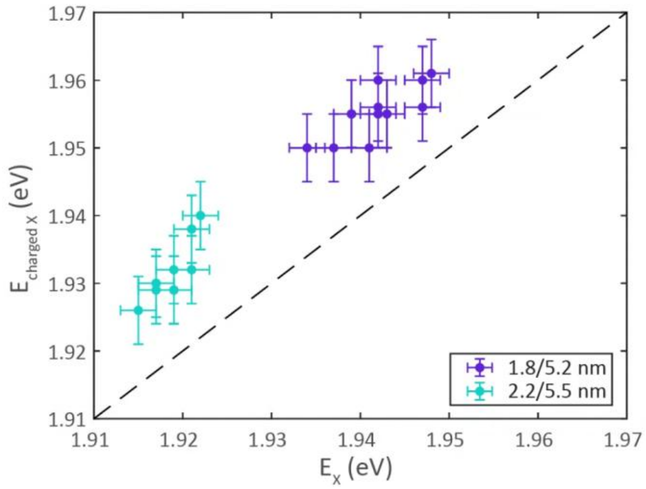

Figure S13. Charged exciton energies. Summary of the charged X energies extracted from the BX analysis (as in Figure 3 in the main text and Figure S12a, b) for the 1.8/5.2 and 2.2/5.5 nm QDs at $\langle N \rangle \sim 0.1$. All charged X energies are blue shifted relative to the neutral X energies (and thus appear above the dashed diagonal line, which is used as a guideline to equal X and charged X energies).

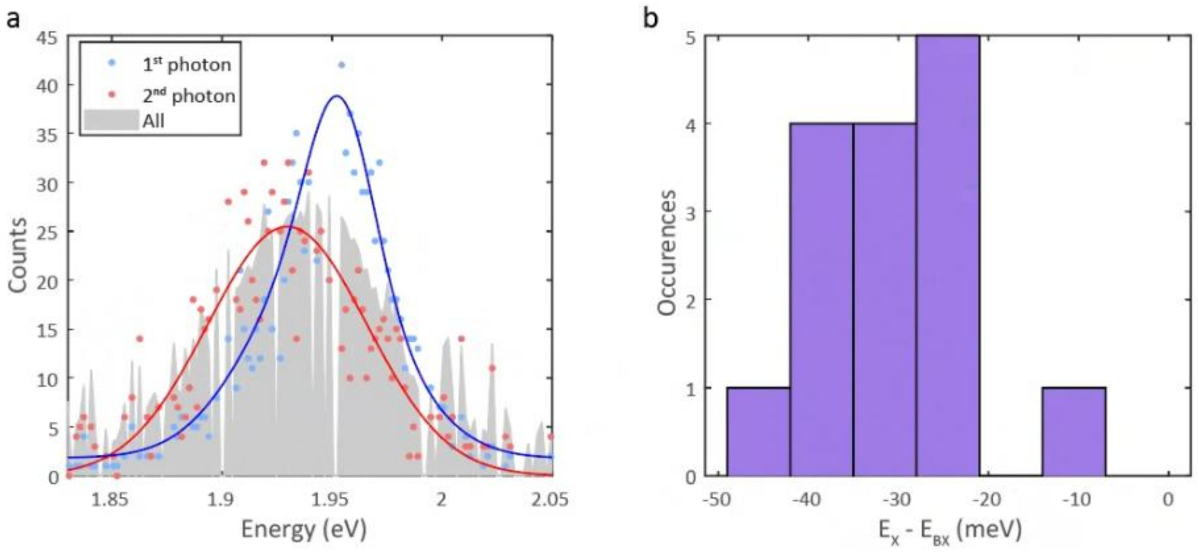

Figure S14. Biexciton binding energy of single 1.8/5.2 nm QDs. (a) An example of heralded spectroscopy analysis in a single QD. The blue dots represent the first photons in the pairs and the pink dots represent the second photons in the pairs. They are both fitted to a multi-gaussian model (in blue and red, respectively). The gray area is a normalized spectrum of all photon events. The difference between the X and BX peaks is -22.5 meV. (b) A histogram of the BX binding energy, measured for 15 single QDs. The average BX binding energy is -31±9 meV.

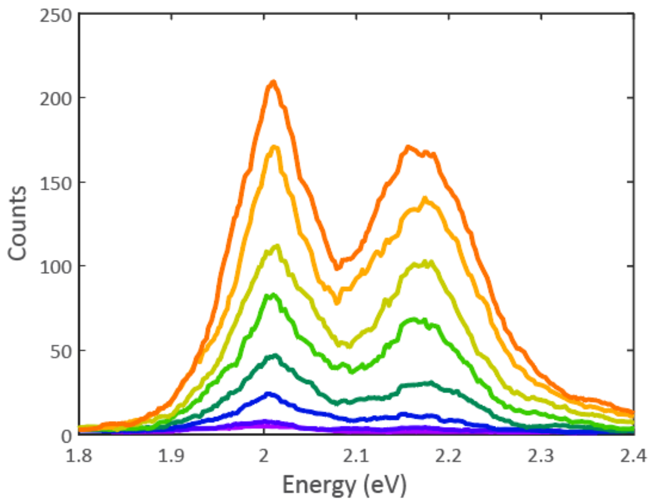

Figure S15. The buildup of the spectrum of the first photons within the photon triplet events in time bins of 20 ps, going from purple to orange (followed by the decay of the spectrum in Figure 5c in the main text).

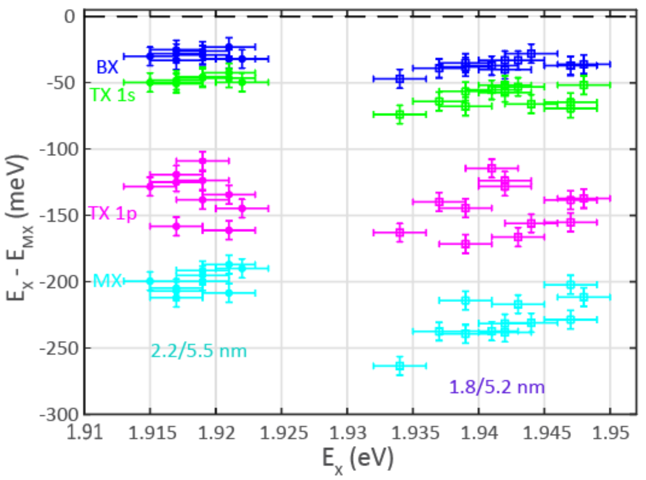

Figure S16. Size dependent multiexciton energies. The difference between the peak energy of the X and that of the BX (in blue), the two triexciton (TX) states (in green and magenta), and the high-order MX (in cyan), for all the ensembles from QDs of 2.2/5.5 nm (on the left side) and of 1.8/5.2 nm (on the right side). A negative value indicates a blue shifted state relative to the X energy. The aggregates from the smaller QDs (2.2/1.5, 1.4/2.3 nm) did not exhibit additional emitting states, aside from the BX. This is due to the strong volume dependence of the Auger recombination rate, which significantly lowers the quantum yield of the multi-excited states in the smaller QDs, rendering them hard to detect even in ensemble spectroscopy.[6,7] In the QDs of 2.2/5.5, 1.8/5.2 nm the trend of the BX binding energies persists

in the higher order emitting states, such that the energies of the states in the smaller 1.8/5.2 nm QDs are slightly higher than in the bigger 2.2/5.5 nm QDs, due to the size-dependent quantum confinement effect.[8]

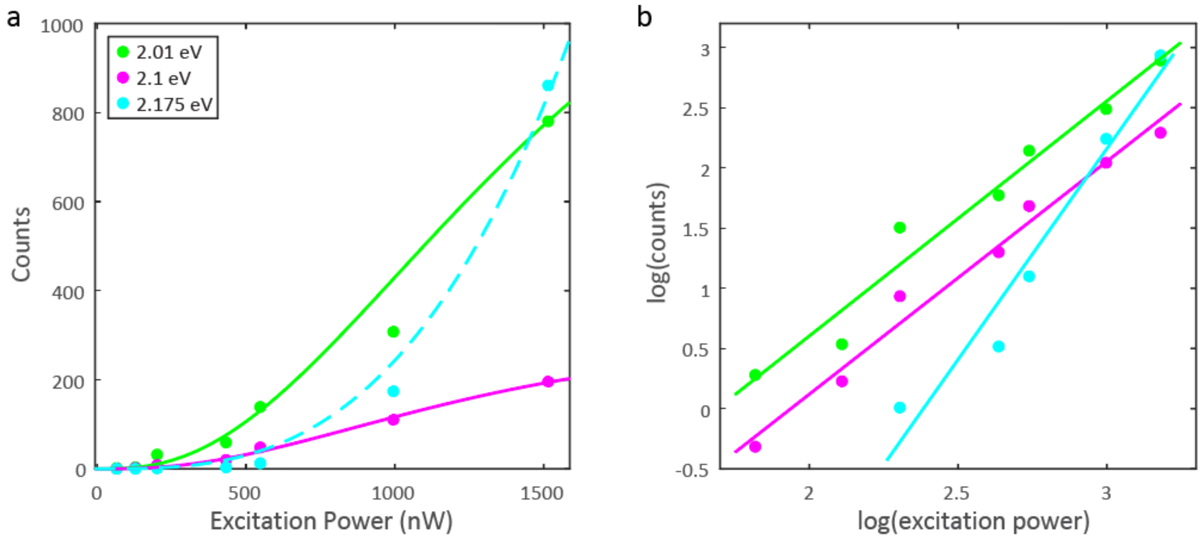

Figure S17. Power dependent triexciton emission. (a) The integrated area under each of the three higher energy gaussian components, as presented in Figure 5a in the main text, versus the excitation power. Each curve is fitted to the probability to generate at least three Xs.[9] The curves that describe the power dependence of the 2.01, 2.1 eV (TX) components fit well to the probability to generate at least three Xs, whereas the component at 2.175 eV (high order MX) showcases a different behavior and does not fit well to this function. The different behavior of the 2.175 eV component becomes clearer in a log-log plot of the integrated area under the gaussian fits and the excitation power, as presented in (b). The two lower energy components (2.01 eV in green and 2.1 eV in magenta) exhibit similar slopes (2.0±0.2 and 1.9±0.2, respectively), whereas the 2.175 eV component in cyan exhibits a significantly higher slope (3.5±0.7), suggesting higher order of MX emission.

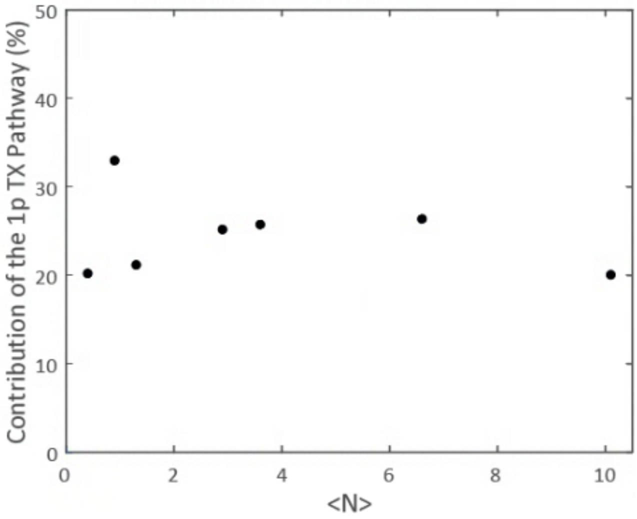

Figure S18. The observed relative contribution of the $1p$ TX pathway to the TX emission signal versus the average number of generated Xs per QD per pulse, $\langle N \rangle$. This figure reflects the independence between the contribution of the $1p$ TX emission and the excitation fluence.

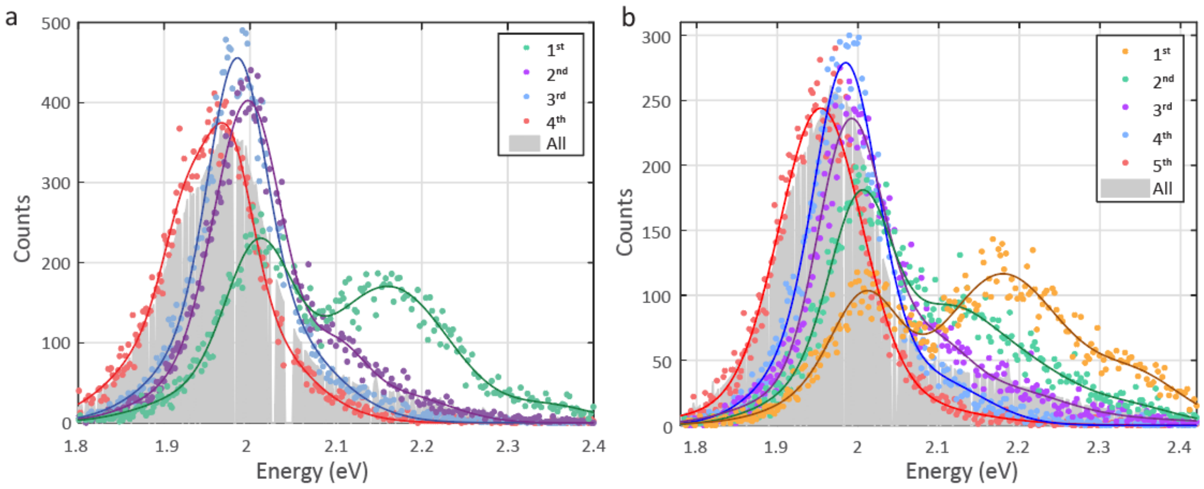

Figure S19. Heralded spectroscopy analysis for four and five sequential photon events. This analysis is for the same ensemble of QDs as in Figure 1h-j, 2-3, 5 in the main text, at $\langle N \rangle = 7 \pm 2$ (a) and $\langle N \rangle = 10 \pm 2$ (b). (a) Spectra of events where four photons are detected after an excitation pulse. The spectrum of the first, second, third, and fourth photons are in green, purple, blue, and pink, respectively. The top gates of arrival time of the first, second, third, and fourth photons are 0.2, 1, 3, and 200 ns. (b) Spectra of events where five photons are detected after an excitation pulse. The top gates of arrival time of the first, second, third, fourth, and fifth photons are 0.3, 0.3, 2, 3, and 200 ns, respectively. The temporal gap between the photon events are 0.8 ns for the 10 nearest neighbor pixels, to avoid cross-talk detection. In

farther pixels, the temporal gap is 0.1 ns (in the 5 photon events, the gap between the first and second photons is even smaller, 0.05 ns). See the Experimental section in the main text, which explains the considerations in applying these temporal conditions.

Section S2. Supplementary text

Temporal conditions in the time gated TX analysis: Figure 5 in the main text presents the time gated TX analysis. The time gates are as follows: 1. First photon (panel d in the main text) – time gate of 0-2 ns in bins of 20 ps. The time gates for the second and third photons are 3 and 200 ns, respectively. 2. Second photon (panel f in the main text) – time gate of 0.4-20 ns in bins of 20 ps. The time gates for the first and third photons are 0.2 and 200 ns, respectively. 3. Third photon (panel h in the main text) – time gate of 1-20 ns in bins of 50 ps. The time gates for the first and second photons are 0.2 and 3 ns, respectively. The temporal gaps between the photons are 0.8 ns, as explained in the Experimental section in the main text. The time gated spectra in the upper panels (c, e, g) only showcases part of this data to demonstrate the spectral changes. Therefore, the presented time ranges are shorter and in panels e and g the time bins are larger (as stated in the main text).

Table S1. Extracted lifetimes from the time-gated TX analysis in Figure 5 in the main text.

	First photon (panel d)*	Second photon (panel f)	Third photon (panel h)
Charged exciton	-**	6.1±0.2 ns (0.861)	6.3±0.5 ns (0.840)
Biexciton	1.4±1.3 ns (0.895)***	1.5±0.1 ns (0.983)	1.54±0.05 ns (0.961)
$1s$ triexciton	323±5 ps (0.992)	428±3 ps (0.986)	520±20 ps (0.899)
$1p$ triexciton	280±30 ps (0.975)	360±10 ps (0.858)	-

*The panels refer to Figure 5 in the main text. **Missing lifetimes are due to low contribution of these states to the spectrum. ***R^2 values.

Extracting the lifetimes of the TX pathways: The observed lifetimes for the two TX components were 323±5 and 280±30 ps. These lifetimes reflect the overall rate of the TX transition, and averaging them yields a lifetime of ~300 ps. This lifetime can be expressed as:

$$\tau_{observed} = \frac{1}{k_{1s} + k_{1p}}$$

Where $k_i = 1/\tau_i$ are the rate constants of the i TX component ($i = 1p$ or $1s$). Additionally, the $1p$ transition contributed to 25% of the TX emission. Accordingly,

$$\phi_{1p} = \frac{k_{1p}}{k_{1s} + k_{1p}}$$

Where ϕ_{1p} is the $1p$ emission fraction.[10] These equations yield the lifetimes of the $1s$ and $1p$ TX transitions: 0.4, 1.2 ns, respectively.

References

[1] Y. S. Park, A. V. Malko, J. Vela, Y. Chen, Y. Ghosh, F. García-Santamaría, J. A. Hollingsworth, V. I. Klimov, H. Htoon, *Phys Rev Lett* **2011**, *106*, 187401.

[2] C. D. M. Donegá, R. Koole, *J Phys Chem C* **2009**, *113*, 6511-6520.

[3] M. Nirmal, B. O. Dabbousi, M. G. Bawendi, J. J. Macklin, J. K. Trautman, T. D. Harris, L. E. Brus, *Nature* **1996**, *383*, 802-804.

[4] C. Galland, Y. Ghosh, A. Steinbrück, M. Sykora, J. A. Hollingsworth, V. I. Klimov, H. Htoon, *Nature* **2011**, *479*, 203-207.

[5] S. Koley, J. Cui, Y. E. Panfil, Y. Ossia, A. Levi, E. Scharf, L. Verbitsky, U. Banin, *Matter* **2022**, *5*, 3997-4014.

[6] V. I. Klimov, A. A. Mikhailovsky, D. W. McBranch, C. A. Leatherdale, M. G. Bawendi, *Science* **2000**, *287*, 1011-1013.

[7] I. Robel, R. Gresback, U. Kortshagen, R. D. Schaller, V. I. Klimov, *Phys Rev Lett* **2009**, *102*, 177404.

[8] D. Norris, M. Bawendi, *Phys Rev B Condens Matter* **1996**, *53*, 16338-16346.

[9] J. M. Caruge, Y. Chan, V. Sundar, H. J. Eisler, M. G. Bawendi, *Phys Rev B Condens Matter* **2004**, *70*, 085316.

[10] K. E. Shulenberger, S. C. Coppieters'T Wallant, M. D. Klein, A. R. McIsaac, T. Goldzak, D. B. Berkinsky, H. Utzat, U. Barotov, T. Van Voorhis, M. G. Bawendi, *Nano Lett* **2021**, *21*, 7457-7464.
